# Jidong Restless Legs Syndrome Cohort Study: Objectives, Design, and Baseline Screening

**DOI:** 10.3389/fneur.2021.682448

**Published:** 2021-10-13

**Authors:** Shu-Hong Wang, Xue-Yu Chen, Xiao-Ping Wang

**Affiliations:** ^1^Department of Neurology, Tongren Hospital, Shanghai Jiao Tong University School of Medicine, Shanghai, China; ^2^Department of Biostatistics, Public Health Institute, Shandong First Medical University and Shandong Academy of Medical Sciences, Shandong, China

**Keywords:** restless legs syndrome, cardiovascular, cerebrovascular, prevention, cohort study

## Abstract

**Background:** Restless legs syndrome (RLS) is a common neurological disorder with unpleasant leg sensations and serious negative effects on mental and physical health. Many observational studies showed that people with RLS had a high risk of vascular diseases, including cerebrovascular and cardiovascular diseases (CVD), but the findings were conflicting. The Jidong RLS Cohort Study is a prospective cohort study designed to mainly examine whether or not RLS is associated with an increased risk of CVD.

**Methods and Design:** The study recruited 8,867 healthy participants older than 18 years from October 2014 to December 2015. Participants received a physical examination in the Staff Hospital, Jidong Oilfield Branch, China National Petroleum Corporation. Baseline data and blood samples were collected. Restless legs syndrome was assessed using the international RLS diagnostic criteria. All of subjects would be followed up until December 2025. Major cardiovascular/cerebrovascular events including cardiac death, myocardial infarction, ischemic heart disease, heart failure, atrial fibrillation, ischemic, and hemorrhagic stroke will be the primary outcomes. Secondary outcomes include all-cause mortality, the decline in quality of life, cognitive impairment, and depression.

**Discussion:** This study will contribute to the scientific evidence on the association between RLS and cardiovascular risks and also provide an unprecedented opportunity for early detection and prevention of CVD.

## Introduction

Restless legs syndrome (RLS), also known as Willis–Ekbom disease (WED), is a common neurological disorder characterized by an uncontrollable urge to move the legs and usually accompanied or caused by uncomfortable and unpleasant sensations. It occurs or worsens during periods of rest or inactivity, and disappears with the movement of the legs (1). Restless legs syndrome is diagnosed according to the International Restless Legs Syndrome Study Group's (IRLSSG) essential diagnostic criteria (2). The reported prevalence of RLS in Asian population is much lower than that in Europe and North America ranging from 2 to 10% (3–7). Women had a one-fold higher risk of developing RLS than men. The mechanism for RLS development still remains to be unclear, although dopaminergic dysfunction and abnormal brain iron metabolism are considered to be the most essential factors for RLS (8).

People living with RLS are more prone to experience an increased risk of cardiovascular diseases including stroke (CVD) and other chronic conditions such as cognitive impairment and depression, mainly due to insufficient sleep and disruption in normal sleep cycle (9–12). Emine et al. demonstrated that RLS is strongly associated with non-dipping hypertension, which has been shown to be an independent predictor of cardiovascular risk (13). Some of the studies found that people with RLS have an elevated risk of stroke and coronary artery disease than those without RLS, whereas others failed to identify such findings (14–16). In addition, there is also evidence that RLS can lead to several vascular disorders including increased aortic stiffness and heart rate variability (17, 18).

Although RLS has been widely studied in many different populations, emerging evidence has also shown that impaired sleep patterns were related to a high risk of CVD and metabolic diseases. However, there still lacks substantial evidence to support that RLS could lead to the development of CVD or other disorders. Moreover, data on the association between RLS and CVD in China are still lacking. In the present study, we designed the Jidong RLS Cohort Study, a large community-based prospective cohort study, to assess the relationship between RLS and CVD. The rationale, design, methods, and baseline characteristics of Jidong RLS Cohort Study were described in the study.

## Methods

### Study Design and Population

**Figure 1:**
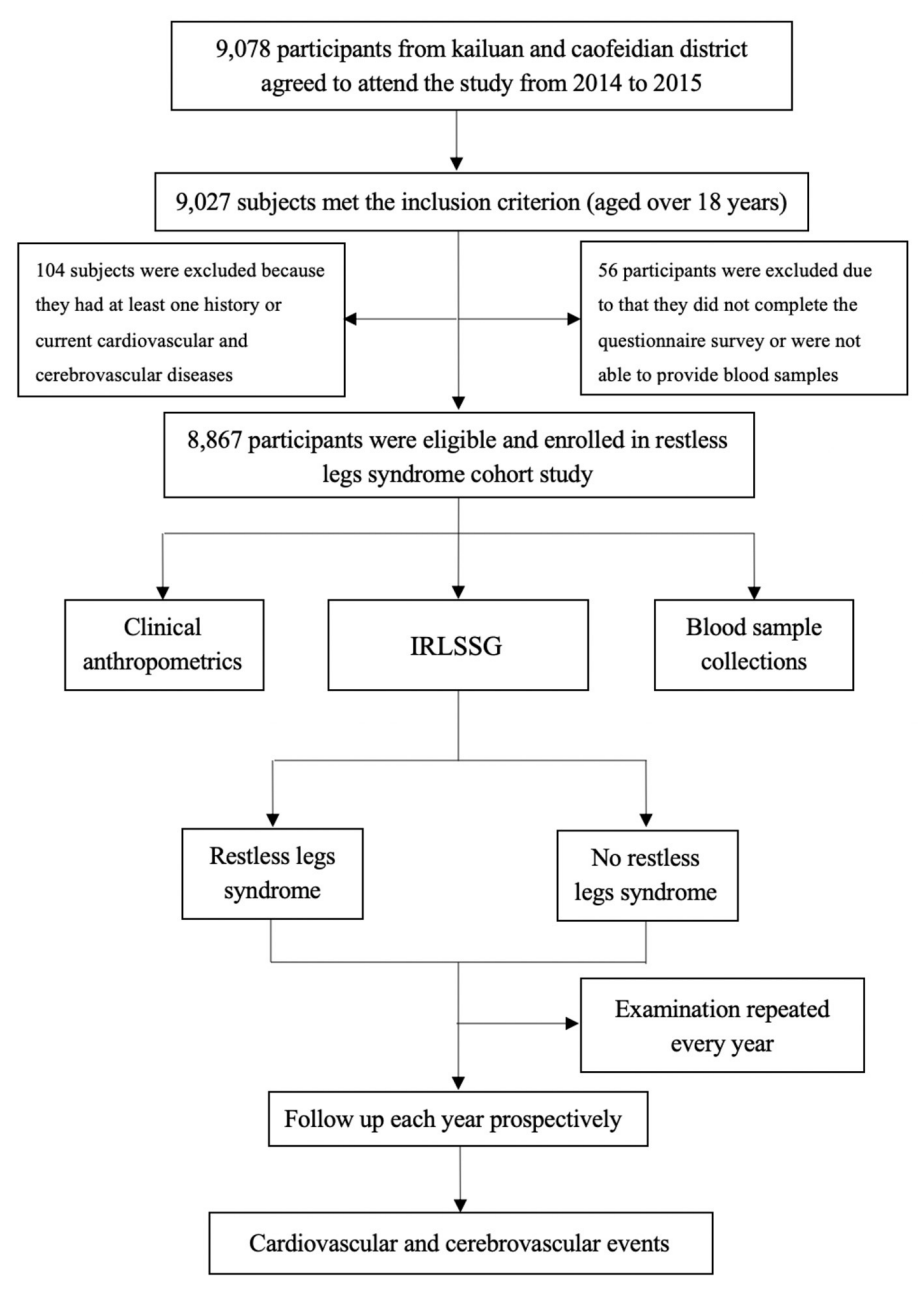
Flow chart of the Jidong Restless Legs Syndrome Cohort Study. IRLSSG, International Restless Legs Syndrome Study Group.

The Jidong RLS Cohort Study is a community-based prospective cohort study designed to investigate the associations between RLS and the risks of CVD. The participants were recruited from Kailuan and Caofeidian communities in Tangshan City, Hebei Province, China. Tangshan is a coastal, modern industrial city located in the central section of Bohai Bay region with a century-long history. It covers an area of more than 10,000 km^2^square kilometers and has a population of 7.03 million including an urban population of 2.93 million. Caofeidian is located in the south of Tangshan City, with an area of 1,944 km^2^ and a population of 2.687 million in 2012. From Oct 2014, we enrolled a total of 8,867 subjects aged over 18 years based on inclusion and exclusion criteria (Figure 1). In this study, all participants were evaluated at baseline and divided into two groups according to IRLSSG diagnostic criteria: the RLS group and the no RLS group.

The study was performed according to the guideline of the World Medical Association Declaration of Helsinki Ethical Principles for Medical Research Involving Human Subjects (2013). Approval was obtained from the Ethics Committee of Kailuan General Hospital of Tangshan City and the Medical Ethics Committee, Staff Hospital, Jidong Oilfield Branch, China National Petroleum Corporation, on July 12, 2013 (approval No. 2013 YILUNZI 1). Written informed consent was obtained from all participants.

### Inclusion Criteria

The inclusion criteria of the study were as follows: aged ≥18 years old, available diagnostic questionnaire on RLS, signed informed consent provided, and blood sample data for the corresponding questionnaire investigation available.

### Exclusion Criteria

The exclusion criteria of the study were as follows: prior history of cardiovascular/cerebrovascular diseases, including coronary heart disease, myocardial infarction, stroke, transient ischemic attack, atrial fibrillation, heart failure, heart valvular diseases, history of any type of cancer, and unavailable questionnaire.

### Data Collection

Data collection was conducted by research assistants and study investigators, who has been trained in measurement procedures and data management program. All data will be saved safely on a unified medical record system with complete confidentiality. In order to ensure privacy respect during the data collection processes, all participants of this study were cited with a code different from their real names.

### Questionnaire Investigation

All questionnaires were performed annually. All participants were asked to complete the health information questionnaire with the assistance of a well-trained research assistant. The questionnaire collected the following information: demographics, lifestyle, and medical history (Table 1). Demographics included age, sex, level of education, and previous history of diseases. Questionnaire of lifestyle included items on smoking, drinking, salt intake, and physical activity. Physical activity was categorized as “inactive,” “moderately active,” or “very active” using the short form of the International Physical Activity Questionnaire (IPAQ). The smoking status was classified as “never,” “current,” or “former” according to self-reported information. Salt intake was classified as low (salt: <6 g/day), medium (salt: 6–10 g/day), or high (salt: >10 g/day). The drinking status was classified as never (no drinks), moderate (less than two standard drinks per day), or heavy (equal or more than two standard drinks per day). One standard drink equals 120 ml of wine or 360 ml of beer or 45 ml of Chinese liquor. Medical histories on hypertension and cardiovascular/cerebrovascular conditions (coronary heart disease, myocardial infarction, stroke, transient ischemic attack, atrial fibrillation, heart failure, and heart valvular diseases) were also collected by questionnaires.

**Table 1:** Testing program in the Jidong Restless Legs Syndrome Cohort Study.

**Test**	**Components**
Specimen collection	Fasting blood sample
Anthropometry	Height, weight, ankle–branchial index, waist and hip circumference
Participant break	Refreshment break with food provided
Cardiovascular	Carotid profiling (blood pressures and pulse wave velocity), transcranial Doppler, and carotid artery sonography, 12-lead electrocardiogram, vascular artery sonography
Respiratory	Obstructive spirometry
Skeleton bone	Density examination
Gynecology	Gynecologic examination, pap smear, ultrasound, pelvic
Restless legs syndrome	International Restless Legs Syndrome Study Group

Restless legs syndrome was diagnosed based on the International RLS Study Group (IRLSSG) diagnostic criteria (Chinese version) (2). Essential diagnostic criteria included: “(1) an urge to move the legs, usually accompanied or caused by an uncomfortable sensation in the legs, (2) beginning or worsening of symptoms during periods of rest or inactivity, (3) partial or total relief of symptoms by movement, and (4) symptoms that are worse in the evening or night compared with during the day or that occur only in the evening or night.” Participants who responded positively to all four questions underwent a face-to-face interview with a trained and experienced neurologist to exclude any mimicking diseases.

The decline in quality of life was assessed using the Short Form-36 Health Survey Questionnaire (SF-36). Short Form-36 Health Survey Questionnaire included eight dimensions: physical functioning, role-physical, role-emotional, social functioning, mental health, bodily pain, general health, and vitality. The final measures were expressed in two summery scores indicating the physical and the mental health-related quality of life, and higher scores represented better health.

The Mini-Mental State Examination (MMSE) was a global cognitive screening instrument that measures cognitive functions, including orientation, memorizing, focus, calculation, deferred recreation, naming, executing spoken commands, writing, and copying. Cognitive impairment was evaluated using the Chinese version of the MMSE, which had performed well in Chinese clinical studies. The MMSE contained 30 questions, each of which was scored as 0 or 1. Maximum score was 30 points. Participants with an MMSE score of ≥24 were considered as having a normal cognitive function. MMSE scores of 20–23 indicated MCI, MMSE scores of 10–20 indicated moderate cognitive impairment, and <10 points indicated dementia.

The Center for Epidemiological Studies-Depression Scale (CES-D) was widely used to evaluate the frequency and severity of depressive symptoms. The CES-D was a 20-item self-report questionnaire that has been shown to have adequate internal consistency and concurrent validity. Each item was rated on a Likert scale from 0 to 3 points (0 = rarely or none of the time/<1 day a week, 1 = sometimes/1–2 days a week, 2 = always or half of a week/3–4 days a week, and 3 = most or all of the times/5–7 days a week). The total score ranges from 0 to 60, with higher scores indicating more severe depressive symptoms. A score of 16 was suggested as the optimal cutoff point in the community setting. Because the Jidong RLS Cohort Study was a community-based epidemiological sample, we used the standardized cutoff of ≥16.

The sleep quality was evaluated by the Athens Insomnia Scale (AIS) with eight items. The possible range of the total score is 0–24. Participants with a score over six points were defined as with insomnia.

### General Physical Examination

General physical examination was performed annually. Body weight (kg) and body height (cm) were measured according to standard anthropometric techniques. Body mass index (BMI) was calculated as body weight (kg) divided by the square of height (m) (kg/m^2^), and the BMI was classified as “ <18.5,” “18.5–23.9,” “24–27.9,” “≥28.” Blood pressure was determined using an electrosphygmomanometer. Three readings of systolic blood pressure and diastolic blood pressure were taken to calculate the mean value as the final blood pressure value. Hypertension was defined based on the following information alone or in combination: (1) as presence of a history of arterial hypertension; (2) using antihypertensive medication; or (3) a systolic blood pressure ≥140 mmHg, or a diastolic blood pressure of ≥90 mmHg.

Blood samples were obtained from the antecubital vein in the morning under fasting conditions using vacuum tubes containing EDTA (ethylene diamine tetraacetic acid) and coagulation-promoting tubes. Fasting blood glucose was measured using the hexokinase/glucose-6-phosphate dehydrogenase method. Cholesterol and triglyceride concentrations were determined with enzymatic methods (Mind Bioengineering Co. Ltd., Shanghai, China). Biochemical markers including blood glucose, serum creatinine, cholesterol, triglyceride, high-density lipoproteins (HDL-C), and low-density lipoproteins (LDL-C) were measured using an autoanalyzer (Hitachi, Tokyo, Japan) at the central laboratory of the Staff Hospital of Jidong Oilfield of China National Petroleum Corporation. Finally, the blood samples were processed and separated on site and stored in a biospecimen bank at −80°C.

### Follow-Up and Outcome Assessment

All participants will be followed up by face-to-face interviews every year in a routine medical examination up to December 31, 2025 or up to the occurrence of an outcome event as defined in the study. The every-5-year incidence of outcomes will be recorded. Data on RLS, demographics, lifestyle, history of disease, basic physical symptoms, blood samples, anthropometry, and the occurrence of other diseases such as CVD were collected by well-trained research assistants and physical examiners. Questionnaires and clinical physical examinations were performed according to the same criteria as the baseline survey. Data on clinical outcomes were collected using a standard operational procedure follow-up system, which included hospitalization reports and files from local general practitioners and medical specialists. Besides, data of the registry on all death cause of participants were obtained from municipal health authorities in Tangshan City for further study.

The primary outcome was major cardiovascular/cerebrovascular events including cardiac death, myocardial infarction, ischemic heart disease, heart failure, atrial fibrillation, ischemic, and hemorrhagic stroke. These events were diagnosed by study physicians and medical experts in the field according to the International Classification of Diseases, 10th edition (ICD-10). The secondary outcomes included all-cause mortality, the decline in quality of life, cognitive impairment, and depression. All-cause mortality was obtained from death certificates and hospital medical records and verified by two senior doctors. The causes were coded according to the ICD-10. The decline in quality of life was assessed using SF-36. Cognitive impairment was evaluated using MMSE and depression evaluated with CES-D.

### Quality Control

All research assistants, interviewers, and physical examiners were well-trained in all items including questionnaires and all aspects of measurements (using standardized techniques). Trainings were conducted on site and within the laboratories of each of the participating investigator under the supervision of experienced staff, until the required standard of testing and competency has been achieved. During the follow-up, regular central monitoring was also undertaken to assess the distribution of certain key variables, the time delay with blood processing, and consistency of the data collected. On-site monitoring visits were undertaken every 6 months by staff from the Hospital of Jidong Oilfield of Chinese National Petroleum.

### Statistical Analysis

All the data will be examined to ensure the accuracy and completeness before statistical analysis and analyzed using SAS software (version 9.1; SAS Institute, Cary, NC, USA). Data with normal distribution will be presented as mean ± SD (standard deviation), and non-normally distributed data will be presented as median and range (min, max). Categorical variables were presented as counts (percentage). The parameters will be compared between the groups, using either Student's *t*-test or Wilcoxon test for continuous variables. The chi-squared test will be used for the comparison of categorical variables. Logistic regression analysis will be used to analyze the associations between life-related cardiovascular risk factors and RLS with odds ratio (OR) and 95% confidence interval (CI). The level for statistical significance will be set at α = 0.05 (two tailed).

## Discussion

Cardiovascular diseases continues to be the leading health problem and major cause of mortality, morbidity, and disability in China and worldwide (19, 20). Some epidemiological studies have provided evidence that possibly links RLS to CVD or with their risk factors such as hypertension and impaired quality of life (21–23). A recent study showed that women with physician-diagnosed RLS had a higher risk of developing coronary heart disease. It also revealed that long duration of the symptoms of RLS was an important factor for the elevated risk (14). Winkelman et al. demonstrated that both RLS and periodic leg movements were associated with incident myocardial infarction in a large sample of older men with 8-year follow-up (24). However, other studies did not identify RLS as an independent risk factor for CVD (25). Findings were inconsistent possibly due to race/ethnicity diversity. So, it is of great significance to investigate the relationship of risk factors with RLS in China. To date, the Jidong RLS Cohort Study is an exclusive population-based cohort of people with RLS, recruited from the general population of mainland China. This study will attempt to explore the possible association between RLS and CVD or their risk factors and provide a new strategy for early detection and prevention of CVD.

There are several RLS studies of clinical trials. Characteristics on design and study population of these studies are presented in the Table 2, and the similarity and differences between our study and others were also shown. Compared with previous studies, our study has several strengths. First, the sample size of our study is larger, and the follow-up time will be longer. Second, our study population includes both male and female over the age of 18, which could help better understand the relationship between RLS and CVD over a wider range of lifetime. Third, RLS mimics were carefully excluded in this study to improve the diagnostic accuracy. Last, our questionnaire investigations were conducted by well-trained investigators through face-to-face interview, and follow-up surveys will be conducted every year. Our study also had some limitations. First, the study sample was from Jidong Oilfield community in northern China, which might not fully represent the entire Chinese population or lack of generalizability to other populations. Second, we had no information on the severity and duration of RLS symptoms. Third, the questionnaire might lead to recall bias. To avoid or minimize recall bias, we could evaluate the reliability of self-reported information by comparing baseline survey and follow-up reports.

**Table 2:** General characteristics of studies on the Restless Legs Syndrome Study.

**Author**	**Sample size**	**Mean age (years)**	**Diagnostic tools**	**Outcome**	**Duration of follow-up (years)**	**Cohort location**
Li et al. (14)	70,977	67	Physician diagnosis	Fatal CHD and first MI event	6	USA
Winter et al. (25)	19,182	66.6	IRLSSG	Major cardiovascular disease and first MI event	7.3	USA
Lin et al. (26)	1,093	63.6	IRLSSG	CVD and death	3.7	Taiwan
Mallon et al. (27)	5,102	46	Upsala Sleep Inventory	All-cause mortality	20	Sweden
Wang et al. (28)	8,867	41.8	IRLSSG	CVD, depression, all-cause mortality	10	China

*IRLSSG, international restless legs syndrome study group; CHD, coronary heart disease; CVD, cardiovascular disease and stroke; MI, myocardial infarction*.

In conclusion, the study described the theoretical objectives, design, and baseline characteristics of Jidong RLS cohort study. This study is a large cohort study on potential associations between RLS and CVD in China, which would be expected to provide more evidence for the intervention of relevant diseases in the future.

## Study Status

The study is ongoing and has completed participant recruitment at the time of submission.

## Ethics Statement

The studies involving human participants were reviewed and approved by Ethics Committee of Kailuan General Hospital of Tangshan City and the Medical Ethics Committee, Staff Hospital, Jidong Oilfield Branch, China National Petroleum Corporation on July 12, 2013 (approval no. 2013 YILUNZI 1). The patients/participants provided their written informed consent to participate in this study.

## Author Contributions

S-HW, X-YC, and X-PW participated in the design and/or optimization of the Jidong RLS protocol. S-HW and X-PW contributed to conception of the study, drafting the manuscript, and critique. X-YC contributed to the data acquisition, manuscript review, and critique. All authors read, approved the final manuscript.

## Funding

This study was supported by the National Natural Science Foundation of China (81671103#).

## Conflict of Interest

The authors declare that the research was conducted in the absence of any commercial or financial relationships that could be construed as a potential conflict of interest.

## Publisher's Note

All claims expressed in this article are solely those of the authors and do not necessarily represent those of their affiliated organizations, or those of the publisher, the editors and the reviewers. Any product that may be evaluated in this article, or claim that may be made by its manufacturer, is not guaranteed or endorsed by the publisher.
